# The Creative Benefits Scale as a Predictor of Attitudes About Aging in Middle-Aged and Older Women

**DOI:** 10.1093/geroni/igaa057.1458

**Published:** 2020-12-16

**Authors:** Sarah Israel, Carolyn Adams-Price

**Affiliations:** 1 Mississippi State University, Starkville, Mississippi, United States; 2 Mississippi State University, Mississippi State, Mississippi, United States

## Abstract

Expectations about aging are connected with both positive traits (e.g., wisdom) and negative traits (e.g., physical frailty). Adams-Price et al. (2016) created the Personal Longevity Scale (PLS), which measures positive expectations (Hope) and negative expectations (Dread). The purpose of this study was to examine how psychological benefits gained from participating in creative hobbies may positively impact middle-aged and older women’s attitudes about aging. In addition, we examined if participant age moderated this relationship. A total of 198 women, aged 50 to 82 years old, completed the Personal Longevity Scale (Hope and Dread subscales) and the Creative Benefits Scale (CBS; used to measure psychological benefits that people may experience as a result of long-term participation in a creative hobby). The CBS includes four subscales: gaining a sense of identity (Identity), getting a sense of relaxation (Calming), feeling closer to God or nature (Spirituality), and receiving recognition from others for one’s hobby (Recognition). Single moderation models suggest that higher Identity scores were linked to more positive attitudes about aging but did not significantly predict negative attitudes. Age provided a marginally significant moderation effect to this relationship such that older women who received more of a sense of identity from their hobby reported the most positive attitudes about aging. Spirituality was linked to more positive attitudes about aging but did not significantly predict negative attitudes. Neither Calming nor Recognition were predictive of either positive or negative attitudes about aging. Implications for women’s development will be discussed from an Eriksonian perspective.

